# Gene Profiling of Bone around Orthodontic Mini-Implants by RNA-Sequencing Analysis

**DOI:** 10.1155/2015/538080

**Published:** 2015-02-11

**Authors:** Kyung-Yen Nahm, Jung Sun Heo, Jae-Hyung Lee, Dong-Yeol Lee, Kyu-Rhim Chung, Hyo-Won Ahn, Seong-Hun Kim

**Affiliations:** ^1^Department of Orthodontics, School of Dentistry, Kyung Hee University, 26 Kyunghee-daero, Dongdaemun-gu, Seoul 130-701, Republic of Korea; ^2^Department of Maxillofacial Biomedical Engineering and Institute of Oral Biology, School of Dentistry, Kyung Hee University, 26 Kyunghee-daero, Dongdaemun-gu, Seoul 130-701, Republic of Korea; ^3^Department of Life and Nanopharmaceutical Sciences, Department of Maxillofacial Biomedical Engineering, School of Dentistry, Kyung Hee University, 26 Kyunghee-daero, Dongdaemun-gu, Seoul 130-701, Republic of Korea; ^4^Department of Orthodontics, Postgraduate School of Dentistry, Ajou University, 164 Worldcup-ro, Yeongtong-gu, Suwon 443-380, Republic of Korea

## Abstract

This study aimed to evaluate the genes that were expressed in the healing bones around SLA-treated titanium orthodontic mini-implants in a beagle at early (1-week) and late (4-week) stages with RNA-sequencing (RNA-Seq). Samples from sites of surgical defects were used as controls. Total RNA was extracted from the tissue around the implants, and an RNA-Seq analysis was performed with Illumina TruSeq. In the 1-week group, genes in the gene ontology (GO) categories of cell growth and the extracellular matrix (ECM) were upregulated, while genes in the categories of the oxidation-reduction process, intermediate filaments, and structural molecule activity were downregulated. In the 4-week group, the genes upregulated included ECM binding, stem cell fate specification, and intramembranous ossification, while genes in the oxidation-reduction process category were downregulated. GO analysis revealed an upregulation of genes that were related to significant mechanisms, including those with roles in cell proliferation, the ECM, growth factors, and osteogenic-related pathways, which are associated with bone formation. From these results, implant-induced bone formation progressed considerably during the times examined in this study. The upregulation or downregulation of selected genes was confirmed with real-time reverse transcription polymerase chain reaction. The RNA-Seq strategy was useful for defining the biological responses to orthodontic mini-implants and identifying the specific genetic networks for targeted evaluations of successful peri-implant bone remodeling.

## 1. Introduction

The development of temporary skeletal anchorage devices (TSADs) in orthodontics has made the mechanics of treatment simpler and more effective than conventional techniques [1–3]. Orthodontic mini-implants, which are generally made of a titanium alloy, are cylindrically shaped with a diameter of 1.5–2.0 mm and a length of 6.0–9.0 mm [4]. Orthodontic force is loaded on the day of mini-implant insertion because primary retention is obtained by mechanically locking the implant onto the cortical plate of the alveolar bone. Thus, cortical bone thickness is the most important factor in determining the initial stability of mini-implants [5].

Previous survival analyses of orthodontic mini-implants showed that the failure rate is greater than 10% [6–8]. The known risk factors for failure are root proximity, insertion site, and inflammation of the peri-implant soft tissue. Many attempts have been made to increase the survival rate of mini-implants [1–3, 9]. Studies have shown that the healing period between mini-implant insertion and orthodontic force application is not critical. Deguchi et al. [10] applied orthodontic force after different healing times (3 weeks, 6 weeks, or 12 weeks) in a dog model, and they found no significant differences in the survival rates. Lee et al. [11] reported that a mini-implant placement angle of less than 60° reduces stability when orthopedic forces are applied in various directions. The design and surface treatment of the mini-implants have been modified similar to those of dental implants, and Kim et al. [12] introduced a two-component mini-implant with a sand blasted with large grit and acid-etched- (SLA-) treated surface.

The key to the success of dental prosthetic implants is osseointegration, and several efforts have been made to increase the area of direct contact between bone and the implant surface [13]. The introduction of a proteoglycan and glycosaminoglycan complex at the interface between the titanium implant and the mineralized tissue was reported to increase the mechanical interlocking and biological interfacial adhesion* in vitro* [14]. Various tools and techniques have been used to investigate the mechanisms of osseointegration. Numerous histologic and histomorphometric analyses and microscopic studies have been performed to examine the bone-implant contact ratio. Transmission electron microscopy, scanning electron microscopy, electron energy loss spectroscopy, and scanning transmission electron microscopy techniques have been introduced to evaluate the titanium-bone interface [15–17].

At the molecular level, only a few transcriptional profiling studies have been performed to characterize osseointegration or alveolar bone remodeling after dental implant placement, and most of these studies have used microarrays. Kojima et al. [18] evaluated gene expression in bone healing around titanium implants in rats with microarray. Ivanovski et al. [19] reported that the transcriptional profile of osseointegration after implant insertion in humans predominantly involved genes that are related to the immune response and extracellular matrix (ECM) formation.

In the field of orthodontics, no studies have examined mini-implant-induced gene expression patterns. The bones healing around orthodontic mini-implants are thought to have unique osteogenic characteristics. Ogawa and Nishimura [20] evaluated the gene expression patterns around implants that have two different surfaces with real-time reverse transcription-polymerase chain reaction (RT-PCR). In the bone surrounding the dual-acid etched surfaced implant, the levels of gene expression of osteonectin and osteocalcin were upregulated compared to their expression in the bone surrounding the turned implant, and bone sialoprotein II, collagen III, and integrin-related genes were upregulated 1 week after implantation [20].

Recently, a technique that combines an RNA sequencing (RNA-Seq) analysis with functional gene classification by whole transcriptome screening has been introduced as an alternative to microarray analysis. Detailed and deep profiling of the transcriptome is possible with this tool [21, 22]. The RNA-Seq analysis can detect a very small amount of RNA compared to that detected by microarray, and the RNA-Seq results show a high level of reproducibility [23]. In addition, fusion gene candidates and insertion and deletion candidates can be identified with RNA-Seq [24]. Moreover, novel genes that have not been annotated in the gene database can be detected [25]. Xu et al. [26] compared transcriptome profiles that were generated by Illumina RNA-Seq and Affymetrix microarray platforms, and their results suggested that RNA-Seq is more advantageous for detecting genes with low-level expression compared with microarrays.

The aim of this study was to use an RNA-Seq analysis to evaluate gene expression in healing bones around titanium orthodontic mini-implants at early and late stages. The molecular mechanisms of bone remodeling around mini-implants were compared with those of bone healing around surgical defects.

## 2. Materials and Methods

### 2.1. Animal Subjects and Implantation

C-implants (1.8 mm × 8.5 mm; Dentium Co., Ltd., Suwon, Korea) were used as SLA-treated titanium implants. Six C-implants were inserted into the basal bone of the mandible in a 12-month-old male beagle dog (10 kg body weight). One single animal was optimal for this study in order to exclude individual genetic differences between samples. A manually drilled osteotomy was followed by manual insertion of the implant rather than machine drilling. Therefore, a saline injection for cooling was not needed. All SLA-treated surfaces were submerged under the bone, and tooth roots or nerves were not injured.

C-implants were implanted under general anesthesia with an intramuscular injection of Zoletil 50 (1.5 cc; Virbac, Carros, France) and Rompun (0.7 cc; Bayer Korea, Ltd., Seoul, Korea) 1 and 4 weeks before euthanization. After the surgery, the antibiotic gentamicin (Komipharm International Co., Ltd., Shinheung, Korea) and the anti-inflammatory agent ketoprofen (Uni Biotech Co., Ltd., Chungnam, Korea) were administered by intramuscular injections. As a control, two surgical defects were created with the same manual drill 1 week before euthanization in the mandibular basal bone. There was no contact between the titanium implant and the bone in the control group.

Samples were retrieved after soft tissue was removed with a trephine bur with an inner diameter of 6.0 mm. The study protocol was approved by the Institutional Animal Care and Use Committee of Kyung Hee University (KHMC-IACUC-2012-026).

### 2.2. RNA Preparation and Quality Check

After the implant and remaining soft tissue were carefully removed, RNA was prepared from the samples with TRIzol reagent (750 *μ*L per sample; Life Technologies, Grand Island, NY, USA) and a RNeasy mini kit (QIAGEN Inc., Valencia, CA, USA). Hard tissue was homogenized with a TissueLyser (QIAGEN Inc.) at 25 Hz for 10 min. The plate was incubated at room temperature and then centrifuged at 12,000 ×g for 1 min. Chloroform (150 *μ*L) was added to the well, and the plate was then vortexed extensively. The plate was incubated at room temperature for 2-3 min and then centrifuged at 12,000 ×g for 1 min. The supernatant (350 *μ*L) was mixed with an equal volume of 70% ethanol in a new well. Then, the RNA was extracted with the RNeasy mini Kit according to the manufacturer's instructions. RNA integrity was measured with an Agilent 2100 Bioanalyzer (Agilent Technologies, Santa Clara, CA, USA), and a sample with an RNA Integrity Number greater than or equal to 8 was considered acceptable.

### 2.3. Transcriptome Sequencing

Transcriptome Sequencing was initiated by transforming the mRNA in the total RNA samples into a template library, which was followed by cluster generation with the components provided in the TruSeq Sample Preparation RNA Kit (Illumina, Inc., San Diego, CA, USA). First, the poly-A-containing mRNA molecules were purified with magnetic beads that were attached to poly-T oligo indicators. Subsequently, at an increased temperature, the mRNA was fragmented into small parts with divalent cations. The cleaved RNA fragments were transcribed into first-strand cDNA with reverse transcriptase and random primers. Subsequently, the second strand was synthesized with RNase H and DNA polymerase. The cDNA fragments were end-repaired by adding a single A base, to which adapters were ligated. Then, the cDNAs were refined and enriched. On the surface of the flow cell, a distinctive bridged amplification reaction occurred with the Illumina kit. Therefore, a flow cell containing numerous unique clusters was placed into the HiSeq 2000 (Illumina, Inc.), which enabled imaging and extension in automated cycles. Sequences were produced by 100-bp paired-end technology. All four nucleotides needed to be present in each sequencing cycle, and this resulted in higher accuracy than the other methods that use only one nucleotide at a time in the reaction mix. The cycles were repeated one base at a time, and a series of images, each of which represented a single-base extension at a unique cluster, was generated.

### 2.4. Gene Ontology (GO) Enrichment Analysis

GO term annotations for* Canis familiaris* were obtained from Ensembl (release 75) at Biomart (http://www.ensembl.org/biomart/martview). In order to determine whether a GO term was enriched in a set of genes (upregulated or downregulated), the number of genes within a set that had a specific GO term was compared to the number in the control gene set. The control gene set was generated by randomly choosing genes from all of the annotated genes (gene length-controlled). Therefore, each test gene had a corresponding control gene. The *P* value for each enriched GO category in the test gene set was calculated as the fraction of times that the *F* test was lower than or equal to the *F* control, where *F* test and *F* control represent the fraction of genes in the test set or random control set, respectively, and were linked with the present GO term based on 10,000 randomly chosen control sets. In order to choose significantly enriched GO terms, a *P* value threshold (1/total number of GO terms considered) was applied.

### 2.5. Real-Time RT-PCR

The samples used in the RNA-Seq analysis were quantified by real-time RT-PCR. cDNA was synthesized with 100 ng of RNA and the Superscript II RT-PCR System (Life Technologies) at 42°C according to the manufacturer's recommendations for oligo(dT)_20_-primed cDNA synthesis. Then, the cDNA was diluted 1 : 2 prior to RT-PCR.

For the quantitative TaqMan PCR, the reactions were performed in 384-well microtiter plates in a final volume of 10 *μ*L with a QuantStudio 12 K Flex Real-time PCR System (Life Technologies). Optimum results were obtained with the following reaction conditions: 5 *μ*L of Universal Master Mix (containing dNTPs, MgCl_2_, reaction buffer, and AmpliTaq Gold; Life Technologies), 90 nM of primers, and 250 nM of fluorescence-labeled TaqMan probe (Life Technologies). Finally, 2 *μ*L of template cDNA was added to the reaction mixture. The primer/TaqMan probe combinations were designed based on each target sequence. The amplification cycling conditions were as follows: a 10-min template denaturation step at 95°C, which was followed by 40 cycles of 15 s at 95°C and 1 min at 60°C. All of the samples were amplified in triplicate, and the data were analyzed with Sequence Detector software (Life Technologies).

The comparative Ct method was used for relative quantification. The target genes were angiopoietin-4 (*ANGPT4*), platelet-derived growth factor receptor *α* (*PDGFRA*), phosphatidylinositol-5-phosphate 4 kinase type-2 *α* (*PIP4K2A*), and WNT1-inducible-signaling pathway protein 2 (*WISP2*).

## 3. Results

### 3.1. Sample Analysis for Quality Control

**Figure 1:**
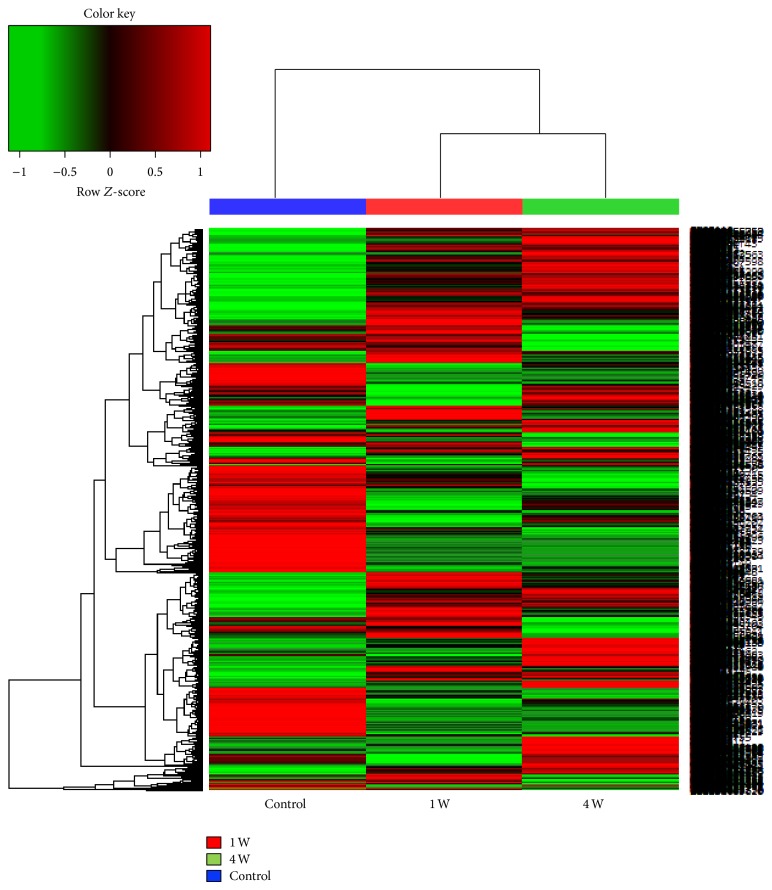
Heatmap of the samples. The red lines represent upregulated genes, and the green lines represent downregulated genes at 1 week and 4 weeks after implantation.

In the first step of the principal gene analysis, 13,690 (of the 21,744 total) transcripts with zero fragments per kilobase of exon per million fragments mapped (FPKMs) were excluded. Of the remaining 8,054 transcripts, 1,643 and 1,839 transcripts had a >2-fold difference in gene expression in the tissues around the implant at 1 week and 4 weeks, respectively, compared with the control surgical defect sites (Figure 1). Among the differentially expressed genes, 773 transcripts were upregulated and 870 were downregulated at 1 week, whereas 937 transcripts were upregulated and 902 were downregulated at 4 weeks.

### 3.2. Functional Annotation of the Transcriptome

Based on the GO classifications in the implant group compared with the surgical defect group at the defined time points of 1 week or 4 weeks after implantation, the principally regulated functions were determined, including the GO terms, gene numbers, and the *P* values. Briefly, in the 1-week implant group, the upregulated GO categories included cell growth (*CYR61*,* IGFBP6*,* LOC476202*,* ESM1*,* IGFBP2*,* CRIM1*, and* WISP2*), DNA helicase activity (*MCM4*,* MCM2*,* MCM6*,* MCM5*,* RECQL*, and* MCM3*), and calcium ion binding (*CCBE1*,* SMOC1*,* EHD2*,* CLEC3B*,* LOC100856635*, and* FKBP10*). The downregulated GO categories included the oxidation-reduction process (*FMO3*,* AHCYL2*,* LOC484867*,* ALDH3A1*,* GSR*, and* FTH1*), intermediate filaments (*KRT6A*,* KRT86*,* KRT18*,* KRT6B*, and* KRT14*), and structural molecule activity (*CLDN10*,* KRT13*,* DSP*,* KRT17*,* VAPA*,* OCLN*, and* CLDN1*) (Table 1). In the 4-week implant group, the upregulated GO categories included ECM binding (*DCN*,* VTN*,* SMOC1*,* BGN*,* NID1*, and* FBLN2*), stem cell fate specification (*SOX17* and* SOX18*), and intramembranous ossification (*MMP2*,* MN1*, and* FGF18*), and the downregulated categories included keratin filaments (*KRT6A*,* KRT86*,* KRT6B*, and* KRT14*), oxidoreductase activity (*FAM81A*,* LOC484867*,* GSR*,* GPX2*, and* SOD1*), and superoxide metabolic process (*PCDP1*,* CYB5R4*,* NOXO1*, and* SOD2*) (Table 2). There was some overlap in the differentially expressed genes between the 1-week and 4-week implant groups. There were 299 upregulated genes and 481 downregulated genes at both time points.

### 3.3. Differential Expression of Selected Gene Categories

A number of different gene categories were examined in the RNA-Seq analysis, and four major functional groupings of differentially expressed genes were identified across the time course of peri-implant bone healing, including (1) cell proliferation, (2) ECM, (3) growth factors, and (4) osteogenic-related genes.

#### 3.3.1. Highly Expressed Genes Involved in Cell Proliferation

The genes and signaling pathways that were related to cell proliferation are listed in Table 3. A number of genes were differentially regulated at 1 week and 4 weeks. At 1 week, the levels of expression of* CDK14*,* PLCD1*, and* ZBTB16* were 2-fold higher than those in the control. Similarly, at 4 weeks, the levels of expression of* FGF18*,* HES4*, and* FYN* were 2-fold higher than those in the control.

#### 3.3.2. Genes Related to ECM Pathways

Many of the genes that were related to the ECM are shown in Table 4.* FN1*,* COL1A2*, and* FBN1* were upregulated at 1 week compared to their levels in the control group. In contrast, at 4 weeks, the levels of expression of other genes, such as* LAMB2*,* VTN*, and* MMP11*, were 2-fold higher than those in the control.

#### 3.3.3. Genes Related to Growth Factors

Other gene groups, such as genes that were related to growth factor pathways, including the platelet-derived growth factor (*PDGF*), fibroblast growth factor (*FGF*), transforming growth factor *β* (*TGF-β* ), vascular endothelial growth factor (*VEGF*), and insulin-like growth factor (*IGF*), were analyzed across the two time points with a cut-off of a 2-fold difference (Table 5). The selected genes were upregulated during the early and late stages of the bone healing process.

#### 3.3.4. Identification of the Genes That Were Related to Bone Remodeling Pathways

Finally, the genes that were related to osteogenic-corresponding pathways, including the regulation of osteoblasts or osteoclast proliferation and development, were categorized (Table 6). Groups of genes that were involved in these pathways, including* PTH1R*,* DLX5*, and* SMOC1*, were upregulated at both time points. Moreover, the genes that were differentially expressed at 1 week and 4 weeks were classified according to their osteogenic differentiation pathway, including the TGF-*β*/BMP and Wnt signaling pathways. At both time points, several genes that encoded proteins that were involved in the TGF-*β*/BMP signaling pathway, which is a critical regulator of skeletal development, were identified, including* TGFB1*,* BMP5*,* MSX1*, and* SMAD6*. The Wnt signaling pathways also appeared to play a critical role during the early and late stages of bone healing. Genes belonging to this pathway, such as* WISP2*,* FZD1*,* FZD2*,* LRP1*, and* LRP5*, were upregulated at both time points.

### 3.4. Confirmation of the Differential Expression of Selected Genes by Real-Time RT-PCR

The RNA-Seq results were validated by evaluating the levels of expression of selected genes with real-time RT-PCR analyses. These analyses demonstrated that the selected genes were up- or downregulated in a similar way to those in the RNA-Seq analysis, and the fold changes of four genes (*ANGPT4*,* PDGFRA*,* PIP4K2A*, and* WISP2*) were confirmed with both methods. The expression levels of these genes that were determined by these two methods were generally consistent, indicating that the RNA-Seq data were valid (Table 7).

## 4. Discussion 

Initial stability is important for the good prognosis of dental prosthetic implants and TSADs in orthodontics. Implant design, surface-treatment method, bone-to-implant contact area, and bone quality are related to initial stability. Secondary stability is mainly related to the osteogenesis and osseointegration that occurs on the surface of the bone that is in contact with the titanium. Favorable initial stability between bone and the titanium fixture immediately after its insertion does not guarantee continuous stability under load, such as occlusal forces or orthodontic forces. This is because the bone-healing processes surrounding the titanium implant differ in each individual. In order to understand bone healing around titanium implants, characteristic gene expression at specific time points in the healing process needs to be investigated.

Implant bone healing has been studied by microscopic analyses, histomorphometric evaluations, immunological identifications, and quantitative PCR [17, 27, 28]. The molecular mechanisms underlying implant-bone healing have been examined by microarray and RNA-Seq analyses [29, 30]. Ivanovski et al. [19] used microarray in order to perform transcriptional profiling of the peri-implant bone. Their study showed that the expression of genes that were related to cell proliferation and immunoinflammatory processes dominated on day 4 after insertion of an SLA-treated titanium implant in a human model. In contrast, on day 14, osteogenesis-, angiogenesis-, and neurogenesis-related genes were predominantly expressed.

Peri-implant bone healing is different from normal bone healing [31]. Kojima et al. [18] studied the gene expression patterns around titanium implants with a microarray containing 20,000 rat genes. They showed that 86 genes were upregulated in the implant group compared with the control group at one or more time points (1 week, 2 weeks, or 4 weeks) after implantation. ECM-related genes, bone resorption-related genes, and growth factor-regulating genes were differentially expressed compared to the osteotomy-healing group.

The present study showed upregulation or downregulation in the levels of expression of a set of gene transcripts that were associated with the presence of an implant in bone. Since we intended to classify the gene transcripts that were potentially responsible for the osteogenic aspects of osseointegration, this study focused on the expression of genes that were induced by peri-implant bone healing. The success of a dental implant depends on predictable biological responses to xenobiotic materials, and further studies of these complex cellular and molecular mechanisms are needed to improve clinical outcomes.

RNA-Seq technology provides information on the expression of thousands of genes in a single experiment [32]. The present study used RNA-Seq technology to analyze the gene expression profile during* in vivo* bone healing around implants that were placed in the mandible. This study was designed to study implant-inducible gene expression patterns at two time points: 1 week (early stage) and 4 weeks (late stage) after implantation. When the gene profile of each implant group at each time was compared with that of a surgical defect control group, we found that a number of gene transcripts were highly upregulated only at 1 week, which suggested the importance of these genes at this early stage in the processes of peri-implant bone healing. Genes encoding ECM constituents were upregulated at 1 week. In contrast, genes associated with bone mineralization, ossification, and regulation of stem cell fate were upregulated at 4 weeks. Therefore, these results suggested that the molecular processes of peri-implant bone healing can be characterized by determining the sets of upregulated genes with transcriptional analyses.

Interestingly, among the genes that were downregulated at both time points were the genes that were related to the oxidation-reduction reaction (redox reaction). Redox reactions are known to play an important role in cell proliferation and differentiation, which are the key processes in the construction of new tissues [33, 34]. Consistent with the present results, a previous study showed that manipulation of the cellular redox state, which results in reduced reactive oxygen species levels, enhances tissue production and increases bone mineralization during osteogenesis [35]. Despite the differences in redox potentials under various biological conditions, the present findings suggested that the modulation of redox reactions increased bone mineralization during implant bone healing.

In the present study, the implant-bone relationships of genes that were related to four categories were assessed, including cell proliferation, ECM, growth factors, and osteogenesis, and these processes have been widely identified as biological mechanisms that are related to bone formation. Cell proliferation-related genes were identified at both time points in peri-implant bone healing. As previously mentioned, cell proliferation is a key biological process in tissue development and regeneration [36, 37]. The present study identified several genes, such as* CDK14*,* PLCD1*, and* ZBTB16*, which were upregulated during peri-implant bone healing. It is well known that cyclin-dependent kinase 14, which is encoded by* CDK14*, is a cell cycle regulator, and its upregulation indicates an increased cell proliferation that increased during peri-implant bone healing [38]. PLCD1 (phospholipase C-delta1), which is a key enzyme in phosphoinositide turnover, is involved in a variety of physiological functions [39]. A previous study showed that PLCD1 could be compartmentalized as a function of cell cycle progression [40]. Moreover, zinc finger and BTB domain-containing protein 16 (ZBTB16) is located in the nucleus and is linked to cell cycle progression [41]. Therefore, the upregulation of these potential implant-inducible genes suggested that cell cycle progression was a critical mechanism during peri-implant bone formation.

Many ECM-related genes were upregulated in the implant group up to 4 weeks after implant (late stage), including* FN1* (fibronectin),* COL1A2* (type I collagen),* FBN1* (fibrillin1),* LAMB2* (laminin subunit *β*2), and* VTN* (vitronectin). These genes are known to support mineral deposition and bone formation [42–45]. Among the upregulated groups of genes were bone resorption-related genes, such as matrix metalloproteinases. The balance between the deposition and resorption of bone is crucial for the formation and maintenance of the peri-implant bone mass [46]. Thus, the present study clearly showed that the regulation of ECM genes was involved in the process of implant bone healing.

The present transcriptome analysis also identified various growth factors that were responsive to the titanium implants. Bone tissue repair is known to involve complex biological events that are regulated by a number of cytokines and growth factors, such as PDGF, FGF, IGF, EGF, and VEGF, which induce the migration of osteoprogenitor cells to damaged sites, their subsequent differentiation towards specific cell lineages, their cell proliferation and revascularization, and the production of ECM [47]. In the present study, these growth factor-related genes were upregulated during peri-implant bone healing. Thus, they could be powerful therapeutic agents in bone formation and regeneration.

Finally, the signaling pathways that were associated with osteogenic development were profiled. The various osteogenic genes that were upregulated during peri-implant bone healing represented the biological and biomechanical establishment of osseointegration. The expression of one of these upregulated genes,* PTH1R* (parathyroid hormone 1 receptor), was 20-fold higher at the late stage of implant healing than that in the control. Despite the debate surrounding the effect of PTH/PTH1R on osteogenesis, a previous study showed that a PTH1R signaling agonist promotes osteoblastic bone formation without stimulating bone resorption [48]. In the present study, other genes, including* SMOC1* (SPARC-related modular calcium-binding protein 1), were upregulated at both time points. The protein encoded by this gene was reported to substantially increase the expression of osteoblast differentiation-related genes in bone marrow-derived mesenchymal stem cells [49]. DLX5 (homeobox protein DLX-5) is another transcription factor that is important for osteoblast differentiation and fracture healing [50]. It has been reported that DLX5 increases the expression of the osteogenic transcription factor Runx2 after BMP2 stimulation, indicating that DLX5 is a BMP-responsive transcriptional activator [51]. The TGF-*β*/BMP and Wnt pathways are the major pathways in osteoblast differentiation and bone formation. Previous studies have shown that TGF-*β*/BMP is the most potent regulator of osteogenesis in various cell types and experimental conditions [52–55]. The present study confirmed the overexpression of TGF-*β*/BMP-related gene transcripts at both the early and late stages of peri-implant bone healing. These results suggested that TGF-*β*/BMP signaling contributed to the repair of impaired skeletogenesis during development. Wnt signaling is another potential target mechanism in the regulation of osteogenesis. Studies have shown that Wnt pathway activation increases osteoblast/osteocyte survival and bone regeneration [56, 57]. In the present study, the RNA-Seq analysis identified differentially expressed genes that were involved in Wnt signaling (*WISP2*,* FZD1*,* FZD2*,* LRP1*, and* LRP5*). During implant-responsive osteogenesis, the Wnt1 gene was upregulated concomitant with the overexpression of Frizzled and LRP, which act as specific receptors for Wnt, suggesting the important role of Wnt machinery in peri-implant bone formation [58, 59].

In conclusion, these findings clearly demonstrated changes in the gene transcripts that were related to redox reactions, cell proliferation, ECM regulation, growth factors, and osteogenic-related TGF-*β*/BMP and Wnt signaling during implant-bone healing in a transcriptome analysis. This study only compared the levels of gene transcript expression between surgical defect and peri-implant healing processes, and a comparison of gene modulation that occurs in response to implants with different properties is planned in future studies. The RNA-Seq analysis with functional gene classification provided an understanding of the biological processes and signaling pathways that were involved in peri-implant bone formation. In addition, the biological data obtained here may help predict the outcomes of clinical strategies that are aimed at promoting osseointegration and bone regeneration.

One limitation of this study was that more bone than just the actual interfacial contact area between the titanium implant and the bone was included in order to meet the minimum bone volume required for the RNA-Seq analysis. Furthermore, if additional studies are done on SLA-treated implants compared with smooth-surface implant controls, significant information will be obtained on implant bone healing according to surface treatment for orthodontic procedures that use TSADs.

## 5. Conclusions

Bone healing at 1 and 4 weeks after the placement of orthodontic mini-implants showed characteristic gene expression in RNA-Seq analyses compared with the healing of surgical defects without mini-implants.

1. Gene profiling analyses showed that gene transcripts that were related to redox reactions, cell proliferation, ECM regulation, growth factors, and the osteogenic-related TGF-*β*/BMP and Wnt signaling pathways were significantly changed.
2. Genes encoding ECM constituents were upregulated at the early stage of healing and genes that were associated with bone mineralization, ossification, and the regulation of stem cell fate were upregulated at the late stage of healing.

## Figures and Tables

**Table 1:** Main genes that were up- or downregulated based on the gene ontology (GO) analysis at week 1 compared with the levels of expression in the control.

Upregulated genes			Downregulated genes		
GO category	Numbers of genes	*P* value	GO category	Numbers of genes	*P* value
Cellular component					
Basement membrane	14	<0.0001	Apical plasma membrane	15	<0.0001
Extracellular matrix	38	<0.0001	Glutamate-cysteine ligase complex	2	<0.0001
Extracellular region	53	<0.0001	Hemoglobin complex	2	<0.0001
Extracellular space	47	<0.0001	Intermediate filament	12	<0.0001
Proteinaceous extracellular matrix	17	0.0001	Keratin filament	8	<0.0001

Biological process					
Cartilage morphogenesis	3	<0.0001	Adhesion to symbiont	2	<0.0001
Collagen fibril organization	9	<0.0001	Cellular aldehyde metabolic process	3	<0.0001
DNA replication	13	<0.0001	Cilium movement	4	0.0003
DNA replication initiation	5	<0.0001	Desmosome organization	2	<0.0001
DNA unwinding involved in DNA replication	4	<0.0001	Fucose metabolic process	2	<0.0001
Extracellular fibril organization	3	0.0003	Glutathione metabolic process	9	<0.0001
Extracellular matrix organization	11	0.0003	Male sex differentiation	2	<0.0001
Regulation of cell growth	7	<0.0001	Neutrophil aggregation	2	<0.0001
			Oxidation-reduction process	49	<0.0001
			Positive regulation of cation channel activity	2	<0.0001
			Protein homotetramerization	8	<0.0001
			Regulation of cell death	3	0.0003
			Response to metal ion	2	<0.0001

Molecular function					
Calcium ion binding	42	0.0002	Aldehyde dehydrogenase (NAD) activity	3	<0.0001
DNA helicase activity	6	<0.0001	Aldehyde dehydrogenase [NAD(P)+] activity	2	<0.0001
Extracellular matrix binding	7	<0.0001	Alpha-L-fucosidase activity	2	<0.0001
Extracellular matrix structural constituent	7	<0.0001	Flavin adenine dinucleotide binding	12	<0.0001
Heparin binding	11	<0.0001	Glutamate-cysteine ligase activity	2	<0.0001
Insulin-like growth factor binding	7	<0.0001	Oxidoreductase activity	44	<0.0001
Platelet-derived growth factor binding	5	<0.0001	Oxidoreductase activity, acting on CH-OH group of donors	5	<0.0001
			Oxidoreductase activity, acting on paired donors, with incorporation or reduction of molecular oxygen, reduced flavin or flavoprotein as one donor, and incorporation of one atom of oxygen	5	0.0003
			Structural molecule activity	16	0.0001
			Superoxide dismutase activity	3	0.0003
			UDP-N-acetylmuramate dehydrogenase activity	4	<0.0001

The values were compared according to fold change.

**Table 2:** Main genes that were up- or downregulated based on the GO analysis at week 4 compared with the levels of expression in the control.

Upregulated genes			Downregulated genes		
GO category	Numbers of genes	*P* value	GO category	Numbers of genes	*P* value
Cellular component					
Extracellular matrix	27	<0.0001	Basolateral plasma membrane	12	0.0001
Extracellular region	55	<0.0001	Cell surface	27	0.0002
Extracellular space	50	<0.0001	Cell-cell junction	13	0.0001
Ribosome	38	<0.0001	Glutamate-cysteine ligase complex	2	<0.0001
			Keratin filament	8	<0.0001

Biological process					
Angiogenesis	16	0.0002	Chaperone mediated protein folding requiring cofactor	5	<0.0001
Bone mineralization	6	0.0002	Desmosome organization	2	<0.0001
Cartilage morphogenesis	3	<0.0001	Glutathione metabolic process	8	<0.0001
Heterophilic cell-cell adhesion	5	0.0003	Glycerol-3-phosphate metabolic process	3	<0.0001
Intramembranous ossification	3	0.0001	Male sex differentiation	2	<0.0001
Negative regulation of mesodermal cell fate specification	3	<0.0001	Neutrophil aggregation	2	<0.0001
Regulation of stem cell division	2	<0.0001	Oxidation-reduction process	49	<0.0001
Stem cell fate specification	2	<0.0001	Smooth muscle cell migration	2	<0.0001
Translation	40	<0.0001	Superoxide metabolic process	5	0.0001
			Termination of signal transduction	2	<0.0001
			Ventricular system development	4	0.0001

Molecular function					
Ceramide kinase activity	2	<0.0001	Catalytic activity	52	<0.0001
Extracellular matrix binding	6	<0.0001	Chemokine binding	2	<0.0001
Retinoid binding	3	<0.0001	Glutamate-cysteine ligase activity	2	<0.0001
Structural constituent of ribosome	37	<0.0001	Glycerol channel activity	2	<0.0001
			Interleukin-8 binding	2	<0.0001
			NAD binding	9	<0.0001
			Oxidoreductase activity	44	<0.0001
			Oxidoreductase activity, acting on CH-OH group of donors	5	<0.0001
			Oxidoreductase activity, acting on the aldehyde or oxo group of donors, NAD or NADP as acceptor	7	0.0001
			Structural molecule activity	18	<0.0001
			UDP-N-acetylmuramate dehydrogenase activity	4	0.0001

The values were compared according to fold change.

**Table 3:** Differentially expressed genes associated with cell proliferation.

Gene symbol	Description	1 W	4 W
AIF1	Allograft inflammatory factor 1 isoform 1	2.81	4.23
ANGPT1	Angiopoietin-1 precursor	3.21	2.18
APOA1	Apolipoprotein A-I	2.12	4.42
BLOC1S2	Biogenesis of lysosome-related organelles complex-1 subunit 2	3.35	2.98
CDK14	Cyclin-dependent kinase 14 isoform 3	4.91	1.45
CLEC11A	C-type lectin domain family 11 member A	4.33	5.44
CXCL12	Stromal cell-derived factor 1 precursor	2.52	4.36
DLC1	Rho GTPase-activating protein 7	4.75	2.67
DPT	Dermatopontin	3.84	2.01
FGF18	Fibroblast growth factor 18	2.37	2.81
FYN	Tyrosine-protein kinase Fyn isoform 2	2.05	2.62
HES4	Transcription factor HES-4	2.16	2.18
LECT1	Leukocyte cell-derived chemotaxin 1	3.68	9.13
MAP2K1	Dual specificity mitogen-activated protein kinase 1	2.42	3.64
MSX1	Homeobox protein MSX-1	3.49	2.94
NFIB	Nuclear factor 1 B-type isoform 3	2.73	2.94
PDGFRA	Platelet-derived growth factor receptor alpha	3.94	2.42
PLCD1	1-Phosphatidylinositol-4,5-bisphosphate phosphodiesterase delta-1 isoform 3	4.40	3.63
RBP4	Retinol-binding protein 4	4.08	6.30
SERPINF1	Pigment epithelium-derived factor	4.82	3.27
SMYD2	N-lysine methyltransferase SMYD2	2.29	2.76
TBX2	T-box transcription factor TBX2	2.22	5.48
VEGFC	Vascular endothelial growth factor C	3.00	2.82
VSIG4	V-set and immunoglobulin domain-containing protein 4	4.73	4.18
ZBTB16	Zinc finger and BTB domain-containing protein 16 isoform 2	3.25	5.25

The values were compared according to fold change. W: week.

**Table 4:** Differentially expressed genes associated with the extracellular matrix (ECM) pathway.

Gene symbol	Description	1 W	4 W
BGN	Biglycan precursor	12.26	5.86
COL1A2	Collagen *α*-2(I) chain precursor	9.27	1.28
COL5A3	Collagen *α*-3(28V)29 chain	6.95	7.00
DCN	Decorin precursor	6.23	4.29
DPT	Dermatopontin	3.84	2.01
EFEMP1	EGF containing fibulin-like extracellular matrix protein 1	3.33	2.58
FBLN1	Fibulin-1	3.14	1.53
FBLN5	Fibulin-5	5.21	4.02
FBN1	Fibrillin-1 isoform 3	6.67	−2.52
FMOD	Fibromodulin	3.18	3.05
FN1	Fibronectin	3.59	−1.19
LAMB2	Laminin subunit beta-2	1.21	2.21
LUM	Lumican	6.28	2.55
MMP11	Stromelysin-3 precursor	1.51	2.06
NID1	Nidogen-1 isoform 1	4.87	3.02
OGN	Mimecan	12.21	3.05
PCOLCE	Procollagen C-endopeptidase enhancer 1 isoform 3	3.17	2.00
PDGFRA	Platelet-derived growth factor receptor alpha	3.94	2.42
POSTN	Periostin	5.61	2.39
PRELP	Prolargin	7.41	6.22
SERPINF1	Pigment epithelium-derived factor	4.82	3.27
SMOC2	SPARC related modular calcium binding 2 precursor	7.68	11.27
TIMP3	Metalloproteinase inhibitor 3 isoform 1	4.19	2.08
TNC	Tenascin precursor	6.19	6.87
VTN	Vitronectin isoform 2	1.06	4.33

The values were compared according to fold change. W: week.

**Table 5:** Differentially expressed genes associated with growth factors.

Gene symbol	Description	1 W	4 W
ANGPT4	Angiopoietin-4	4.65	9.81
CLEC11A	C-type lectin domain family 11 member A	4.33	5.44
CLEC3B	Tetranectin	3.31	6.98
CRIM1	Cysteine-rich motor neuron 1 protein	4.27	3.51
EFEMP1	EGF containing fibulin-like extracellular matrix protein 1	3.33	2.58
ESM1	Endothelial cell-specific molecule 1 isoform 2	3.37	7.04
FGF13	Fibroblast growth factor 13 isoform 1	1.67	2.33
FGF18	Fibroblast growth factor 18	2.37	2.81
FGF23	Fibroblast growth factor 23	−1.12	3.93
FYN	Tyrosine-protein kinase Fyn isoform 2	2.05	2.62
IGFBP2	Insulin-like growth factor binding protein 2	2.89	−1.44
IGFBP6	Insulin-like growth factor binding protein 6 isoform 3	3.11	2.45
LECT1	Leukocyte cell-derived chemotaxin 1	3.68	9.13
LRP1	Prolow-density lipoprotein receptor-related protein 1	2.25	3.28
LTBP3	Latent-transforming growth factor beta-binding protein 3	1.76	2.79
NRP2	Neuropilin-2 isoform 4	8.79	3.50
PDGFRA	Platelet-derived growth factor receptor *α*	3.94	2.42
PTN	Pleiotrophin	3.28	2.34
SIRT1	NAD-dependent deacetylase sirtuin-1 isoform 1	2.67	2.93
SMAD6	Mothers against decapentaplegic homolog 6	2.36	6.82
TGFB1	Transforming growth factor beta-1 precursor	4.07	8.44
TWF2	Twinfilin-2	2.49	2.92
VEGFC	Vascular endothelial growth factor C	3.00	2.82
WISP2	WNT1-inducible-signaling pathway protein 2	8.61	6.15
YWHAG	14-3-3 protein *γ*	3.26	2.45

The values were compared according to fold change. W: week.

**Table 6:** Differentially expressed genes associated with osteogenesis.

Gene symbol	Description	1 W	4 W
Osteogenesis			
CYR61	Protein CYR61	3.03	1.67
DLX5	Homeobox protein DLX-5 isoform 2	3.03	9.05
FGF23	Fibroblast growth factor 23	−1.12	3.93
ID3	DNA-binding protein inhibitor ID-3	2.55	3.22
ILK	Integrin-linked protein kinase isoform 1	2.49	1.38
OSR2	Protein odd-skipped-related 2 isoform 1	4.71	1.74
PTH1R	Parathyroid hormone/parathyroid hormone-related peptide receptor precursor	1.95	20.12
SFRP2	Secreted frizzled-related protein 2 precursor	3.34	4.35
SMAD3	Mothers against decapentaplegic homolog 3	2.10	−1.15
SMOC1	SPARC-related modular calcium-binding protein 1 isoform 1	3.83	4.36
SNAI1	Zinc finger protein SNAI1	2.26	2.66
SNAI2	Zinc finger protein SNAI2	2.81	2.61
TYROBP	TYRO protein tyrosine kinase-binding protein precursor	1.44	2.12

TGF-*β*			
BMP5	Bone morphogenetic protein 5 isoform 1	2.68	2.34
BMPER	BMP-binding endothelial regulator protein	1.77	3.14
CYR61	Protein CYR61	3.03	1.67
DLX5	Homeobox protein DLX-5 isoform 2	3.03	9.05
GATA3	Trans-acting T-cell-specific transcription factor GATA-3 isoform 1	2.19	1.01
HES4	Transcription factor HES-4	2.16	2.18
MSX1	Homeobox protein MSX-1	3.49	2.94
SFRP2	Secreted frizzled-related protein 2 precursor	3.34	4.35
SMAD6	Mothers against decapentaplegic homolog 6	2.36	6.82
TGFB1	Transforming growth factor beta-1 precursor	4.07	8.44

WNT			
BARX1	BARX homeobox 1	−1.03	25.85
CDK14	Cyclin-dependent kinase 14 isoform 3	4.91	1.45
DKK1	Dickkopf-related protein 1	12.93	2.09
DLX5	Homeobox protein DLX-5 isoform 2	3.03	9.05
FZD1	Frizzled-1	4.08	2.10
FZD2	Frizzled-2	2.05	1.30
HIC1	Hypermethylated in cancer 1 protein isoform 1	2.62	2.03
LATS2	Serine/threonine-protein kinase LATS2	4.14	2.15
LRP1	Prolow-density lipoprotein receptor-related protein 1	2.25	3.28
LRP5	Low-density lipoprotein receptor-related protein 5	2.89	1.43
NXN	Nucleoredoxin	3.35	2.48
SFRP2	Secreted frizzled-related protein 2 precursor	3.34	4.35
SFRP4	Secreted frizzled-related protein 4 isoform 1	2.90	4.82
SNAI2	Zinc finger protein SNAI2	2.81	2.61
WISP2	WNT1-inducible-signaling pathway protein 2	8.61	6.15

The values were compared according to fold change. W: week.

**Table 7:** Real-time polymerase chain reaction (PCR) validation.

Symbol	Description	Assay ID	Time	RNA-sequencing	Real-time PCR
ANGPT4	Angiopoietin-4	Cf02656885_m1	1 w	2.22	1.76
			4 w	3.29	1.52
PDGFRA	Platelet-derived growth factor receptor alpha	Cf02687293_m1	1 w	1.98	3.39
			4 w	1.27	0.74
PIP4K2A	Phosphatidylinositol-5-phosphate 4-kinase type-2 alpha	Cf02703943_mH	1 w	1.09	1.98
			4 w	1.57	1.20
WISP2	WNT1-inducible-signaling pathway protein 2	Cf02702369_g1	1 w	3.11	1.65
			4 w	2.62	4.06

The values were compared according to fold change.
